# Placebo and nocebo effects of pharmacotherapy for obsessive–compulsive related disorders: a systematic review and meta-analysis

**DOI:** 10.1017/neu.2026.10070

**Published:** 2026-03-26

**Authors:** Jacob Hoffman, Taryn Williams, Dan J. Stein

**Affiliations:** Department of Psychiatry and Mental Health, Neuroscience Institute, Faculty of Health Sciences, University of Cape Town, Cape Town, South Africa

**Keywords:** obsessive–compulsive disorders, placebo effect, nocebo effect, meta-analysis, psychopharmacology

## Abstract

**Objective:**

This systematic review and meta-analysis aimed to quantify the magnitude of placebo and nocebo effects in pharmacological trials for OCRDs and identify clinical and methodological moderators influencing these effects.

**Methods:**

A comprehensive literature search was conducted across multiple databases and clinical trial registries up to May 2025. Randomised, placebo-controlled trials involving pharmacological interventions for OCRDs were included. The primary outcomes were placebo effect size and placebo response rate; secondary outcomes included nocebo response rate and side effect profile. Data were extracted independently and meta-analysed using random effects models. Meta-regression was performed to assess moderators of placebo response.

**Results:**

Fifteen eligible trials (*N* = 640; placebo *N* = 341) were included. The pooled placebo effect size was moderate (SMC = −0.63; 95% CI −0.77 to −0.48), with low heterogeneity (*I* ^2^ = 4.73%). The placebo response rate was 21%, and the nocebo response rate was 18%. Despite testing a broad range of potential moderators, including clinical characteristics, methodological design, and medication class, no significant predictors of placebo effect size were identified. Side effects were reported in nearly one-third of placebo recipients, underscoring the relevance of nocebo effects.

**Conclusions:**

Placebo and nocebo responses are noteworthy in trials for OCRDs and may influence perceived treatment efficacy. Variability in placebo responses is not well explained by currently measurable moderators. Further research is needed to explore neurobiological, psychological, and methodological contributors to expectancy effects in OCRD pharmacotherapy trials.

Summations

- There is a moderate placebo effect size (SMC = −0.63) across pharmacology trials for OCRDs. This is larger than found in OCD, but smaller than in anxiety or mood disorders.
- No significant clinical or methodological moderators of this effect size were identified.
- The pooled dropout rate of 18% and presence of 0.44 side effects reported per participant indicates presence of nocebo effects in pharmacology trials for OCRDs.

Considerations

- Trichotillomania was over-represented in the study sample compared to other OCRDs, and thus these results may not be generalisable to all OCRDs.
- Clinical and methodological moderators of the placebo effect may be investigated more closely with more published trials or using individual participant data.
- Clinician- versus participant-rated scales were not investigated, nor were ITT versus completer analysis. These could add depth to our understanding of placebo and nocebo effects.

## Introduction

The Diagnostic and Statistical Manual of Mental Disorders, Fifth Edition (DSM-5) and the International Classification of Diseases, Eleventh Revision (ICD-11) introduce new sections for obsessive–compulsive and related disorders (OCRDs), a distinct group of conditions characterised by intrusive thoughts, repetitive behaviour and significant distress or functional impairment (American Psychiatric Association, 2013; World Health Organisation, 2019). OCRDs include body dysmorphic disorder (BDD), hoarding disorder (HD), trichotillomania (TTM; hair-pulling disorder), excoriation (skin-picking) disorder (ED or SPD), and other body-focussed repetitive behaviours (BFRBs). These disorders are associated with substantial morbidity, and often require psychotherapeutic and pharmacological intervention (American Psychiatric Association, 2013).

Despite treatment advances, many patients experience only partial remission, and relapse is common (Swedo *et al*., 1993; Keijsers *et al*., 2006). Randomised, placebo-controlled trials remain the gold standard for assessing medication efficacy. Understanding the placebo and nocebo response is vital when interpreting the potential benefit of such interventions (Chavarria *et al*., 2017; Evers *et al*., 2018; Huneke *et al*., 2020; Huneke, 2022).

Placebo response refers to an observed response in the placebo arm of a trial, influenced by natural course, regression to the mean, and trial design features (Evers *et al*., 2018; Huneke *et al*., 2024). These arise as a complex interaction between clinical, methodological, and psychobiological factors (Evers *et al*., 2018; Huneke *et al*., 2020, 2022). Increased placebo response reduces the contrast between active and placebo groups, reducing the ability to detect true drug effects. Similarly, nocebo response refers to adverse effects observed in the placebo arm and may influence tolerability outcomes. Together, placebo and nocebo effects influence observed treatment responses in randomized controlled trials (RCTs) through psychobiological processes that interact with clinical characteristics and methodological features of trials (Evers *et al*., 2018; Huneke *et al*., 2024).

### Placebo response in psychiatry

Participants in RCTs enter the therapeutic environment of any trial with preconceived ideas around the act of taking pills and the therapeutic setting (Leuchter *et al*., 2014). Myriad factors can influence the observed response within the placebo group, affecting the magnitude of differentiation between groups. First, clinical factors may influence the placebo response in psychopharmacology trials, including participants’ age, primary diagnosis, severity of symptoms at baseline, medication class being studied, and presence of comorbid psychiatric conditions (Furukawa *et al*., 2016, 2018; Evers *et al*., 2018; Leucht *et al*., 2018; Motta *et al*., 2023). Second, trial characteristics and methodology have been associated with differences in the observed placebo response, including publication year, study design, risk of bias, number of outcomes measured, number of treatment arms utilised, number of study sites, the presence of a placebo run-in period, and industry funding (Alphs *et al*., 2012; Furukawa *et al*., 2016; Evers *et al*., 2018; Kotzalidis *et al*., 2019; Leucht *et al*., 2018; Motta *et al*., 2023). The impact of these factors on the placebo response carries significant implications for the interpretation of effect sizes observed in RCTs.

A recent umbrella review demonstrated substantial heterogeneity in the placebo effect across multiple psychiatric conditions (Huneke *et al*., 2024), with placebo effect sizes ranging from small in obsessive–compulsive disorder (OCD) (Mohamadi *et al*., 2023), to large in generalised anxiety disorder (Bandelow *et al*., 2015). Several potential moderators were associated with larger placebo response, including later publication year, younger age, more trial sites, larger sample size, increased baseline severity, and larger active treatment effect sizes (Huneke *et al*., 2024). These findings underscore the importance of disorder-specific analyses in psychiatry.

### Placebo response in antidepressant trials

In antidepressant trials, concerns have been raised regarding a perceived increase in placebo response rates over time (Bandelow *et al*., 2015; Furukawa *et al*., 2016, 2018). Some investigations of antidepressants across conditions have indicated a temporal increase in the observed placebo response rate within studies involving SSRIs, whereas others have found little change over time in the placebo response rate (Bandelow *et al*., 2015; Furukawa *et al*., 2016; Kirsch, 2019; Stahl & Greenberg, 2019). Furukawa *et al.*, describe a break point in 1991, after which publication year and placebo response are no longer correlated (Furukawa *et al*., 2018). This contrasts with Bandelow et al., who demonstrate a strong relationship between publication year and placebo response for anxiety disorders across the entire publication period (Bandelow *et al*., 2015). Whether a similar break point exists has not been investigated in OCRDs.

Earlier trials demonstrated lower placebo responses due to methodological factors affecting allocation concealment and outcome assessor blinding, which may amplify effect sizes (Holper & Hengartner, 2020). Older antidepressants may have prominent side effects, which can facilitate unblinding, enabling clinicians to distinguish between placebo and active medication groups. Variations in placebo response across different medications supports this, with those exhibiting more significant anticholinergic or sedative side effects (such as amitriptyline and trazodone) displaying enhanced efficacy and reduced placebo response, suggesting the potential for unmasking of treatment allocation and bias in outcome assessment (Holper & Hengartner, 2020).

Placebo response to better tolerated antidepressant medications (i.e., citalopram, escitalopram fluoxetine, sertraline, duloxetine, and venlafaxine) or atypical antidepressants (i.e., mirtazapine and agomelatine) show an increased placebo response and reduced medication effect size, suggesting difficulty in accurately detecting allocation of participants to active or placebo arms of the trials, and hence better maintenance of blinding in these trials (Holper & Hengartner, 2020). Furthermore, meta-analysis of studies comparing antidepressants to ‘active’ placebo (i.e., with anticholinergic or other effects), demonstrated a diminished effect size of antidepressants, suggesting that the efficacy of antidepressants in trials using inert placebos may be inflated (Moncrieff *et al*., 2004). However, this meta-analysis only included nine studies, and warrants further investigation if robust conclusions are to be drawn (Moncrieff *et al*., 2004; Jørgensen *et al*., 2021; Juul *et al*., 2021).

### Placebo response in OCD and OCRDs

OCD studies have consistently shown a reduced placebo response compared to other psychiatric disorders (Huneke *et al*., 2024). The underlying reasons for this reduced placebo response remain unclear, although they may stem from differences in psychopathology, demographic, and clinical characteristics of the study population or study design in depression, anxiety, and OCD trials (Kotzalidis *et al*., 2019; Mohamadi *et al*., 2023). Despite an overall smaller placebo response rate in OCD, a significant net increase in placebo effect size was observed by Kotzalidis *et al*., from 1991 to 2017, with an increase in placebo response rate from 2010 to 2017 (Kotzalidis *et al*., 2019). The factors driving this are uncertain, however, changes in the studied medications and differences in trial methodology such as treatment duration, baseline symptom severity, multicentricity, trial location, and presence of placebo run-in may be relevant factors (Kotzalidis *et al*., 2019; Motta *et al*., 2023).

First, earlier trials in OCD focused on clomipramine, a tricyclic antidepressant, with notable, common anticholinergic side effects. This may have led to similar hypothesised unblinding as in depression trials of tricyclic antidepressants, although this has not been systematically analysed. Second, as clomipramine was one of the first treatments for OCD, participants were likely treatment-naïve in earlier studies, whereas recent studies in OCD include add-on trials for treatment-resistant populations, thus changes in patient populations and severity of condition at baseline could represent significant confounding factors (Kasper & Dold, 2015; Kotzalidis *et al*., 2019). Third, earlier trials were predominantly conducted in the USA, whereas more recent studies were conducted in multiple countries. Differences in study methodology and clinical characteristics of patient cohorts may similarly influence observed placebo response (Kotzalidis *et al*., 2019).

Longer treatment duration is hypothesised to lead to an increased placebo response, as regression to the mean and spontaneous remission may be interpreted as response to treatment. A few factors are relevant when interpreting the influence of treatment duration on observed placebo response. First, the variance in the natural history of the condition, particularly relevant for OCD and OCRDs, which typically have an exacerbating-remitting course (Perugi *et al*., 1998). Second, shorter studies may also be more vulnerable to random effects or transient effects based on expectancy of improvement (Mohamadi *et al*., 2023).

To date, placebo response in other OCRDs has not been systematically explored through meta-analysis to determine rates of placebo response, or meta-regression to investigate contributing factors. One study examined the placebo response in TTM across five studies, with 104 included participants receiving placebo (Grant *et al*., 2017). In this study, Grant et al., found placebo response rate (31%), similar to that of OCD in children, and adolescents (31%) (Cohen *et al*., 2008), although placebo effect size was not calculated, which limits its comparability to other meta-analyses (Mohamadi *et al*., 2023; Motta *et al*., 2023). No specific clinical or demographic characteristics were identified that delineated responders from non-responders to placebo in these five trials (Grant *et al*., 2017). Thus, the evidence base of the placebo effect in OCRDs remains sparse and warrants further investigation.

### Nocebo effects in OCD and OCRDs

The nocebo effect is defined as the adverse effects experienced when receiving a placebo (Evers *et al*., 2018). Compared to the placebo effect, the nocebo effect has been the focus of much less attention. Multiple factors may influence the development of a nocebo effect, notably an expectation bias attributed to taking medication, or a conditioning response with expectation of pain (Požgain *et al*., 2014).

It is hypothesised that anxiety and negative expectations may contribute to this observed response (Colloca & Benedetti, 2007; Rooney *et al*., 2023). In a meta-analysis of the nocebo effect in medication trials for depression, nearly half of placebo-treated participants had at least one adverse effect or dropped out due to adverse effects. Older age, higher percentage of treatment responders in the active arm and duration of illness were negatively correlated with the nocebo dropout rate (Mitsikostas *et al*., 2014). Whilst the nocebo effect has been investigated in trials of some psychiatric disorders (Mitsikostas *et al*., 2014; Blasini *et al*., 2018; Faasse, 2019; Weimer *et al*., 2020), there are no studies examining the nocebo effect specifically in OCD or any other OCRDs.

## Study rationale

### Study aim

This systematic review and meta-analysis aimed to quantify the placebo effect size, the placebo response rate, and nocebo-related outcomes in pharmacotherapy RCTs for OCRDs. Additionally, clinical, methodological, and pharmacological moderators were evaluated through meta-regression.

### Research questions

1. What is the magnitude of the placebo response rate and placebo effect size in pharmacotherapy RCTs for OCRDs?
2. Which clinical and methodological factors moderate placebo response rate and placebo effect size in OCRDS?
3. What is the dropout rate in placebo groups of OCRD pharmacotherapy RCTs?
4. What is the rate of adverse effects in placebo groups of OCRD pharmacotherapy RCTs?

## Methodology

The study was conducted according to the Preferred Reporting Items for Systematic Reviews and Meta-Analyses statement and was registered with Prospero (CRD42024539956) prior to commencing (Page *et al*., 2021). The study was approved by the University of Cape Town Human Research Ethics Committee (HREC Ref 839/2023).

### Search strategy

PsycINFO, Web of Science, PubMed Central (which includes MEDLINE), and Cochrane Library databases were systematically searched for randomised, placebo-controlled trials on the pharmacological treatment of OCRDs up until 25 May 2025. In addition, ClinicalTrials.gov and the WHO trials portal were searched for published and unpublished trials. Recent systematic reviews for OCRDs were also used to identify relevant RCTs for inclusion in the study. Reference lists of all identified trials were also searched for additional studies. There were no language restrictions applied during the search.

Searches were conducted using the following keywords: ‘placebo’ OR ‘nocebo’ AND ‘obsessive’ OR ‘trichotillomania’ OR ‘compulsive hair-pulling’ OR ‘skin-picking disorder’ OR ‘excoriation’ OR ‘body dysmorph*’ OR ‘hoarding’ OR ‘OCRD’ AND ‘randomized control trial’ OR ‘RCT’. Results were screened using Endnote 20.1 citation manager software (The Endnote Team, 2013). Duplicate studies were removed and articles were screened for inclusion or exclusion. Details of this process are documented using a PRISMA flow diagram.

### Study selection

#### Types of studies

Randomized, placebo-controlled trials investigating pharmacotherapeutic agents for the treatment of obsessive–compulsive related disorders, including trichotillomania, excoriation disorder, BDD, HD, and other body-focused repetitive behaviours were included in the study. Cluster-RCT, crossover trials, and multi-arm trials were also considered for inclusion. Quasi-randomised controlled trials and open-label studies were excluded. Studies conducted in single or multi-centre settings were considered for review, in both inpatient and outpatient settings.

#### Types of participants

Study participants were required to have a primary diagnosis of trichotillomania, excoriation disorder, BDD, HD, or other body-focused repetitive behaviour according to criteria from the Diagnostic and Statistical Manual of Mental Disorders (DSM, version III to V, or similar) (American Psychiatric Association, 2013). There were no restrictions placed on age or gender of the participants. Studies including participants with psychiatric comorbidities were also included, however, if the study included participants with an alternate primary diagnosis, these were excluded.

#### Types of interventions and controls

Participants received treatment with a psychopharmacological medication of any class, or treatment with a standardised, plant-based extract or dietary supplement. Medications with broad psychotropic effects were considered, including dopamine antagonists, ion channel modulators, glutamate modulators, serotonin transporter inhibitors, serotonin, and noradrenaline transporter inhibitors, noradrenaline transporter inhibitors, dopamine transporter inhibitors, noradrenergic, and specific serotonergic modulators, monoamine oxidase inhibitors, and mixed receptor antagonists with serotonin and noradrenaline transporter inhibition. There were no restrictions placed on the timing, duration, and pharmaceutical co-interventions, provided that participants were stable on other psychotropic medication for a minimum of three months prior to the study.

Studies were excluded if participants were treated concurrently with psychotherapy or neurostimulation. If the therapeutic agent was a non-standardised oral agent, such as a non-standardised dietary supplement, these studies were also excluded.

The presence of a placebo arm was required for inclusion in the study, and both active or passive placebos were considered for inclusion. No restriction was placed on timing, dose or duration of the placebo. Those studies without a pharmacological placebo were excluded, including studies using psychotherapy or treatment as usual as a control group. If the study included a separate psychotherapy arm, or treatment as usual arm, in addition to medication and pharmacological placebo arms, these were considered for inclusion.

#### Types of outcome measures

There were two primary outcomes and two secondary outcomes for this study. Placebo response rate and placebo effect size were the primary outcomes, whereas nocebo response rate (dropouts due to any reason) and side effect rates were the secondary outcomes.

Placebo response rate was defined as the percentage of participants in the placebo arm of the included studies responding to placebo determined using the Clinical Global Impressions-Improvement Item (CGI-I, a widely-used scale ranging from 1 = ‘very much improved’ to 7 = ‘very much worse’). This was used as a dichotomous measure of treatment response in which responders are defined as having a change item score of 1 = ‘very much improved’ or 2 = ‘much improved’ (Guy, 1976). Where both dichotomous and continuous data for the CGI-I are presented, only the categorical measure of ‘responders’ versus ‘non-responders’ was included. Where CGI-I was unavailable, a similar measure was considered.

The placebo effect size was defined as the change in symptom severity from baseline to endpoint on the relevant symptom severity scale (e.g., Yale-Brown Obsessive Compulsive Scale (YBOCS), the Massachusetts General Hospital Hair-Pulling Scale (MGH-HPS) or similar, validated scale) for participants in the placebo arm of the trial. For each trial only a single symptom severity outcome scale was analysed, with preference given to a validated scale with good inter-rater reliability, convergent validity and internal consistency, such as the MGH-HPS (Keuthen *et al*., 1995).

The nocebo response rate was measured as the proportion of participants in the placebo arm who withdrew (dropped out) from the study for any reason.

The side effect rate was measured as the proportion of participants in the placebo arm who experience specific side effects. These were reported as rates for each named side effect, as well as the proportion of participants who experienced any side effect.

### Study selection and data extraction

Titles of studies identified in the searches were examined by two reviewers (JH and TW). Following a first round of screening titles and abstract, full text of relevant articles that appeared to meet the inclusion criteria were further reviewed. The full eligibility criteria for inclusion in the review were applied, and any conflicts of opinion were discussed with another review author (DJS). JH and TW then independently extracted data from the included studies. Any disagreements regarding the data extraction were resolved by discussion with a third review author (DJS). Digital data extraction sheets were created for collation and recording of descriptive information, summary statistics of the outcomes, quality scale ratings, and associated commentary. A Google Form was developed to streamline the data extraction process, and entry directly into an Excel spreadsheet (https://forms.gle/ocnYveMgsBfHAwDe6). Following data extraction, spreadsheets were combined and additional spreadsheets generated for use within R for further analysis. Authors of the original publications were contacted by JH for missing information. No reports required translation.

The following information was collected from each study:

1. Details of the trials including first author, year of publication, affiliation, country in which research was conducted, trial protocol ID, number of participating centres, and presence of industry funding (whether the trial or authors were supported by the pharmaceutical industry, and whether medication was provided by the industry).
2. Details of trial methodology including screening tools used, diagnostic criteria employed, number of treatment arms, whether it was a parallel arm or crossover trial, the presence of a placebo run-in, whether a fixed or flexible dosing regime was used, and total duration of the trial.
3. Details of participants including primary diagnosis, number of participants, mean age, gender distribution, baseline symptom severity, and percentage of participants with comorbid depression, anxiety, another OCRD (including OCD), attention-deficit hyperactivity disorder (ADHD), or other psychiatric comorbidities.
4. Details of the intervention and comparator including the name of the medication used, whether the placebo was an active or passive pharmacological agent, duration of treatment and dose of intervention.
5. Details of the outcome measures employed including names of outcome scales used, whether an intention-to-treat (ITT) sample was utilised, dichotomous data regarding treatment response and continuous data regarding changes in symptoms severity, representing dichotomous and continuous measures of efficacy. These data were extracted for both intervention and placebo arms.
6. Details regarding tolerability, including the number of total dropouts per group, the number that dropped out due to side effects, side effects reported and proportion of participants reporting each side effect.
7. Details regarding methodological quality for assessment according to the Cochrane Risk of Bias 2 Tool.

### Assessment of risk of bias in included studies

Two review authors (JH and TW) independently examined the components described in the Cochrane Risk of Bias 2 tool for each included study using a standard form (Sterne *et al*., 2019). This form includes information related to random sequence generation, allocation concealment, blinding of participants, and study personnel, blinding of outcome assessment, incomplete outcome data (attrition bias), selective reporting (reporting bias), and other sources of bias. For each trial, each domain was rated as *low*, *some concerns,* or *high* with regards to risk of bias. Each trial also received an overall rating of *low, some concerns* or *high*. Risk of bias assessments were based on guidelines in the Cochrane Handbook, and results are visually summarised using the *robvis* online tool (McGuinness & Higgins, 2021; Higgins *et al*., 2024).

### Main comparison

Baseline and endpoint symptom severity was compared for participants in placebo groups from trials investigating the following medications for treating OCRDs in adults and children.

- Antioxidants (e.g., silymarin).
- Cannabinoids (e.g., dronabinol).
- Cell signal transducers (e.g., inositol).
- Dopamine antagonists (e.g., olanzapine).
- Glutamate modulators (e.g., N-acetylcysteine).
- Mixed receptor antagonists with serotonin and noradrenaline transporter inhibition (e.g., clomipramine).
- NMDA receptor antagonists (e.g., memantine)
- Opioid antagonists (e.g., naltrexone).
- Serotonin transporter inhibitors (e.g., citalopram, fluoxetine, and sertraline).

### Measures of placebo and nocebo effect

#### Measures of placebo effect size

For continuous measures of the placebo effect, we calculated placebo effect size in each trial as the standardised mean change (SMC) using an outcome scale of interest measuring symptom severity (i.e., MGH-HPS, YBOCS-TTM, or NIMH-TSS). The SMC, related to standardised mean difference (SMD; Cohen’s d or Hedges’ g) (Cohen, 2013), standardises the differences between separate scales employed in the trials and calculates the change between pre- and post-intervention means, accounting for correlation between sample groups. The formula for calculating the SMC and standard deviation of the mean change is found below:

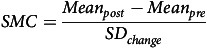




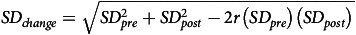




This calculation of SD_*change*_ accounts for an adjustment for correlation (*r*). As the correlation between groups is unknown the *r* value used in calculating the SD_*change*_ was kept as the default of *r* = 0.5.

There is no universally accepted interpretation for the magnitude of SMC, however, as it is related to measures of SMD, similar thresholds based on those for Cohen’s D were used. An SMC of 0.2 was considered a ‘small’ effect size, 0.5 considered a ‘medium’ effect size and 0.8 considered a ‘large’ effect size (Cohen, 2013).

#### Measures of placebo response rate

Placebo response rate was defined as the proportion of participants in the placebo groups who were rated as treatment responders at the end of the trial, based on dichotomous measures of treatment response (i.e., CGI-I or similar). Comparison with the active group was not done. The pooled proportion was calculated through meta-analysis for proportions using the *metafor* package in *R* (Viechtbauer, 2010).

#### Measures of nocebo response rate

Nocebo response rate was calculated as the proportion of participants who dropped out from the trial for any reason. The overall dropout rate was calculated, averaged across all included trials which reported this information.

#### Measures of tolerability

The tolerability of placebo was reported as the proportion of participants receiving placebo who experienced specific side effects. These were averaged as simple proportions across trials which report similar or related side effects. The overall side effect rate was also examined as the proportion of participants in the placebo group who experienced any side effect.

### Unit of analysis issues

Certain study designs, such as those with multiple treatment groups, crossover trials, and cluster randomised trials require specific attention due to unit of analysis issues. There were no studies with multiple treatment groups or cluster randomised trials in this review. Crossover trials were included in the calculation of outcomes of interest when it was possible to extract medication and placebo data from the first treatment period, or when there was a washout period of adequate duration, defined as a minimum of two weeks (or four for fluoxetine, due to its long half-life).

### Dealing with missing data

All analyses were conducted as ITT, testing the effect of assignment to intervention group (Higgins *et al*., 2024). An ITT analysis utilises the total number of participants randomly assigned to the placebo group as the denominator for all calculations regarding placebo effect size or response rate. Only data from trials that provide this information were eligible for inclusion in these analyses.

Where summary statistics were derived using imputation of missing data, preference was given to outcome measures derived from mixed effects models, followed by last observation carried forward models, and last by observed cases. Those derived from mixed effect models are more robust than other alternate imputation methods (Verbeke & Molenberghs, 2009).

### Data synthesis

Continuous measures of placebo effect size from each trial were synthesised through meta-analysis. Random effects meta-analysis of the SMC was conducted using a restricted maximum likelihood (REML) estimator with the R *metafor* package (R Core Team, 2024; Viechtbauer, 2010). The random effects model accounts for within-study sampling error and between-studies variability in calculating the precision of the confidence interval. The REML estimator provides a stable estimate of heterogeneity and between-study variance (*τ* ^2^). The *escalc()* function was used to calculate the SMC and its corresponding variance, following the formula detailed above. The *rma()* function was used to account for heterogeneity among studies. The resultant overall SMC reflects the average placebo effect size, as well as its 95% confidence interval, and measures of heterogeneity. For synthesis of proportions of placebo response, nocebo response, and side effect rates, meta-analysis was conducted in *R* using the *metafor* package and the *rma.glmm()* function, accounting for weighting of the result based on study sample size (R Core Team, 2024; Viechtbauer, 2010). Forest plots, pooled proportions, 95% confidence intervals, and heterogeneity measures were generated.

### Assessment of heterogeneity

For meta-analysis of placebo effect, presence of significant heterogeneity was determined by using the Chi^2^ test of heterogeneity (Cochran, 1954). The presence of heterogeneity was defined as a *P* value < 0.10 (Deeks *et al*., 2024, p. 10). This cutoff was used given the low power of the Chi^2^ statistic when the number of trials is small. In addition, the *I* ^2^ statistic was calculated in *R*, and is used to test the magnitude of observed heterogeneity across studies (Higgins & Thompson, 2002; Higgins *et al*., 2003).

Heterogeneity was interpreted as:

- 0–40%: Might not be important;
- 30–60%: May represent moderate heterogeneity;
- 50–90%: May represent substantial heterogeneity;
- 75–100%: Considerable heterogeneity.

Where significant heterogeneity is found, a visual examination of the forest plot was used to identify studies that may be responsible for the high heterogeneity, and prompted further interrogation regarding the source of the heterogeneity.

### Subgroup and sensitivity analysis

To test the robustness of the findings from meta-analysis of the placebo effect size, and to investigate sources of variance and heterogeneity, subgroup and sensitivity analysis were planned.

We planned subgroup analysis based on presence or absence of placebo run-in, crossover versus parallel arm study design, primary diagnosis, presence of industry funding, and overall risk of bias. Whilst it is recommended that a minimum of 10 studies are required for a viable subgroup analysis (Deeks *et al*., 2024, p. 10), we used a minimum of three studies due to the small number of included trials. Additional subgroup analyses were planned for studies investigating serotonin transporter inhibitors, as the most commonly studied class of medication in OCRDs, and for NAC as the most commonly studied individual agent in the included studies. These subgroup analyses were added post hoc, as compared to the original study protocol, following identification of included studies.

Two sensitivity analyses were conducted to test the robustness of the finding for meta-analysis of placebo effect size. The first used a leave-one-out approach wherein meta-analysis was repeated multiple times, each time omitting one study (Viechtbauer & Cheung, 2010). This evaluates the influence of the omitted study on the overall pooled SMC. Changes in the effect size, confidence intervals, and heterogeneity were examined to determine the influence of each single study. The second sensitivity analysis was conducted for different measures of correlation between pre- and post- measurements, by investigating different values for *r* in the calculation of the SMC. Five values were tested, with *r* = 0.10, 0.25, 0.50, 0.75, and 0.90 successively.

### Univariate and multiple meta-regression analysis

Meta-regression is a useful tool for investigating sources of variance and heterogeneity within a meta-analysis (Deeks *et al*., 2024, p. 10). It is recommended that a minimum of 10 trials are included within the meta-regression, necessitating that each variable of interest has data for each of the 10 trials. There must also be sufficient variability within the possible values of the variable investigated.

Univariate meta-regression analysis was performed to investigate the relationship between placebo effect size and various methodological and clinical variables. Random effects meta-regression was conducted in R using the *metafor* package, with REML estimation and Wald-type *z*-tests. Univariate meta-regression was conducted for year of publication, number of study sites, number of treatment arms, presence or absence of placebo run-in, flexible versus fixed dose schedule, crossover versus parallel arm study design, duration of the trial, number of outcomes measured, number of participants in placebo arm, age of participants, primary diagnosis, percentage of participants with comorbidities (depression, anxiety, other OCRD, OCD, and ADHD), medication class, baseline severity (standardised using a *Z*-score normalisation method) (Cohen *et al*., 2013), active treatment effect size (calculated as SMC), industry funding and overall risk of bias score.

Multiple meta-regression was planned to investigate the relationship between multiple independent variables.

### Assessment of publication bias

Publication bias arises due to the tendency for studies with positive, or statistically significant results, being more likely published. Those with non-significant, negative or inconclusive findings may be less likely to appear in published literature. This results in a bias within the published literature base (Gilbody & Song, 2000). Publication bias was analysed through visual inspection of a funnel plot of placebo effect size and placebo response rate (Deeks *et al*., 2024). Small-study effects were analysed through application of Egger’s test (Egger *et al*., 1997).

## Results

### Results of the search

**Figure 1.**
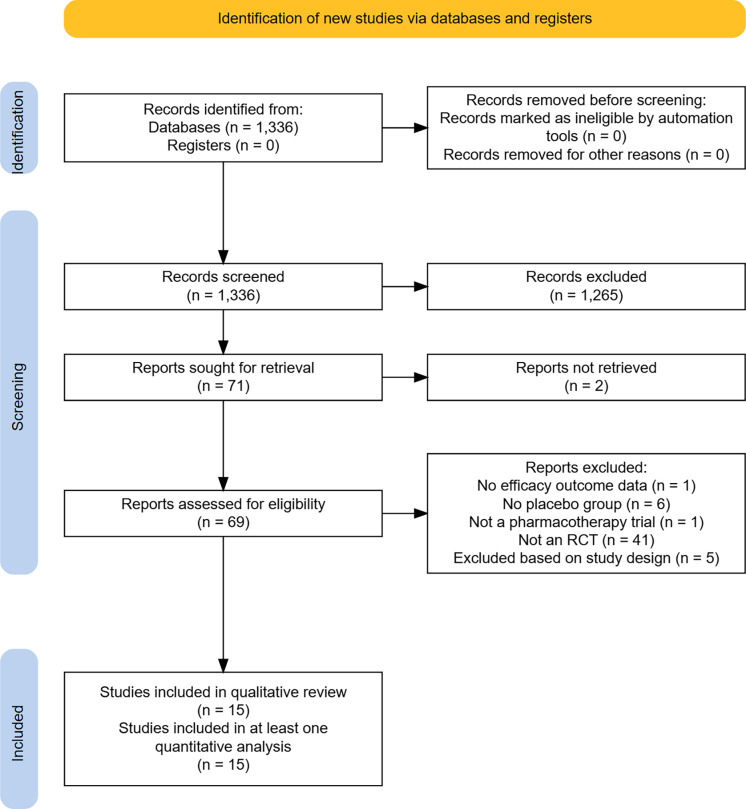
PRISMA flow diagram for search results and study selection process. RCT – Randomised controlled trial.

Details of the literature search process are presented in *Figure 1: PRISMA flow diagram for search results and study selection process*. Initial search identified 1336 after deduplication. After initial screening of titles and abstracts, 71 trials were assessed for inclusion. Following a second screening phase 54 studies were excluded (reasons for exclusion detailed in Figure 1), and 14 trials were identified for inclusion in the review (Bloch *et al.*., 2013; Christenson *et al.*., 1991; Dougherty *et al.*., 2006; Grant *et al.*., 2009, 2014, 2016, 2019, 2022, 2023b; Leppink *et al.*., 2017; Ninan *et al.*., 2000; Phillips *et al.*., 2002; Streichenwein & Thornby, 1995; Van Ameringen *et al.*., 2010). An additional trial was identified for inclusion through searching reference lists (Arbabi *et al*., 2008). These 15 trials were further interrogated, data extracted, and included within further analyses.

### Characteristics of included studies

Summary details for included studies may be found in Table 1: Characteristics of Included Studies.

**Table 1.** Characteristics of included studies

Author (Year)	Number of participants	Diagnosis	Mean age	Design/Weeks	Drug	Primary outcomes	Placebo effect size	Placebo responders
Arbabi (2008)	45 (13 M; 32 F)	SPD	32	4-week parallel arm RCT	Citalopram	VAS	N/a	N/a
Bloch (2013)	39 (5 M; 34 F)	TTM	14	12-week parallel arm RCT	NAC	MGH-HPS	0.67	21% (4/19)
Christenson (1991)	16 (2 M; 14 F)	TTM	32	18-week crossover RCT	Fluoxetine	No. of hair-pulling episodes per week	N/a	N/a
Dougherty (2006)	37	TTM	N/a	12-week parallel arm RCT	Sertraline	MGH-HPS	0.28	N/a
Grant (2009)	50 (5 M; 45 F)	TTM	33	12-week parallel arm RCT	NAC	MGH-HPS	0.18	16% (4/25)
Grant (2014)	51 (7 M; 44 F)	TTM	32	8-week parallel arm RCT	Naltrexone	MGH-HPS	1.52	35% (9/26)
Grant (2016)	66 (8 M; 58 F)	SPD	35	12-week parallel arm RCT	NAC	NE-YBOCS	N/a	19% (4/21)
Grant (2019)	22 (1 M; 21 F)	TTM	29	13-week crossover RCT	Silymarin	NIMH-TSS	0.46	31% (5/16)
Grant (2022)	50 (9 M; 41 F)	TTM & SPD	33	10-week parallel arm RCT	Dronabinol	NIMH-TSS	0.86	50% (8/16)
Grant et al. (2023b)	100 (15 M; 80 F – in analysis)	TTM & SPD	30	8-week parallel arm RCT	Memantine	NIMH-TSS/SPD	0.67	8% (3/36)
Leppink (2017)	38 (3 M; 35 F)	TTM	28	10-week parallel arm RCT	Inositol	MGH-HPS	1.01	32% (6/19)
Ninan (2000)	23 (10 M; 13 F)	TTM	34	9-week parallel arm RCT	Clomipramine	NIMH-TSS	0.25	0% (0/6)
Phillips (2002)	67 (21 M; 46 F)	BDD	32	12-week parallel arm RCT	Fluoxetine	BDD-YBOCS	0.63	24% (8/33)
Streichenwein (1995)	23 (3 M; 20 F)	TTM	39	29-week crossover RCT	Fluoxetine	Severity of hair-pulling	N/a	N/a
Van Ameringen (2010)	25 (8 M; 17 F)	TTM	34	12-week parallel arm RCT	Olanzapine	CGI-I	0.82	17% (2/12)

In ‘participants’ = M, males, F, females. In ‘diagnosis’ = BDD, body dysmorphic disorder, SPD, skin-picking disorder, TTM, trichotillomania. In ‘age’ = N/a, data unavailable. In ‘design’ = RCT, randomised controlled trial. In ‘drug’: NAC, N-acetylcysteine. In ‘primary outcomes’: BDD-YBOCS, Yale-Brown Obsessive Compulsive Scale modified for Body Dysmorphic Disorder, CGI-I, Clinical Global Impressions – Improvement, MGH-HPS, Massachusetts General Hospital Hair-Pulling Scale, NE-YBOCS, Yale-Brown Obsessive Compulsive Scale modified for Neurotic Excoriation, NIMH-TSS, National Institute of Mental Health - Trichotillomania Severity Scale, NIMH-TSS/SPD, National Institute of Mental Health - Trichotillomania Symptom Severity Scale modified for Skin-Picking Disorder, VAS, Visual Analogue Scale. In ‘placebo effect’ and ‘placebo responders’ = N/a, data unavailable. Placebo effect size is represented by Hedges g for pre- to post- symptom severity change. Placebo responders are represented as percentage of those in the placebo group who were classified as treatment responders, no. of responders / total no. of placebo participants shown in parentheses.

#### Design and setting

All of the included studies were RCTs, including a placebo arm. Of the studies, 13 were conducted in the USA, one in Canada (Van Ameringen *et al*., 2010) and one in Iran (Arbabi *et al*., 2008). All studies were conducted in an outpatient setting, and all studies were single-centre trials. Only a single trial included participants below 18 years old (Bloch *et al*., 2013). The other 14 studies were conducted on adults aged 18 years and older. Three of the trials used a crossover design (Christenson *et al*., 1991; Streichenwein & Thornby, 1995; Grant *et al*., 2019). The other 12 studies used a parallel-group design. All studies had two treatment arms, except for one which had three parallel arms (Ninan *et al*., 2000).

The duration of the crossover trials ranged from 13 to 29 weeks (including both arms and a washout period) (*k* = 3, mean = 20 ± 8.19 weeks). The parallel arm trials ranged from four to 12 weeks in duration (*k* = 12, mean = 10.08 ± 2.50 weeks). A placebo run-in was used in three of the studies (Streichenwein & Thornby, 1995; Phillips *et al*., 2002; Dougherty *et al*., 2006). Nine studies used a flexible dosing schedule, whereas the other six used a fixed dosing regimen (Christenson *et al*., 1991; Arbabi *et al*., 2008; Bloch *et al*., 2013; Grant *et al*., 2014, 2019, 2023b). Dosing progression regimens were classified as fixed provided that all participants followed the same regimen, whereas they were classified as flexible if dosing was adjusted based on side effects, symptom improvement, or other factors. All studies were published in English. Six studies provided evidence of industry funding (Streichenwein & Thornby, 1995; Ninan *et al*., 2000; Dougherty *et al*., 2006; Grant *et al*., 2009, 2016; Van Ameringen *et al*., 2010).

#### Sample size and participants

The 15 studies included a total of 640 participants (mean 23.70 ± 10.6) with 341 participants in the placebo arms of the trials (mean 22.73 ± 9.32). The smallest placebo group consisted of six participants (Ninan *et al*., 2000), whereas the largest included 45 individuals (Grant *et al*., 2023b).

Twelve of the 15 trials used inclusion criteria based on DSM-III or later (i.e., The Structured Clinical Interview for DSM-5 or similar), to select participants for the trials (Ninan *et al*., 2000; Phillips *et al*., 2002; Dougherty *et al*., 2006; Grant *et al*., 2009, 2014, 2016, 2019, 2022, 2023b; Van Ameringen *et al*., 2010; Bloch *et al*., 2013; Leppink *et al*., 2017). Two of the studies used the Minnesota Trichotillomania Assessment Inventory for screening of participants (Christenson *et al*., 1991; Streichenwein & Thornby, 1995). A single study utilised a diagnostic measure based on repetitive skin-picking resulting in noticeable tissue damage and associated emotional distress and/or functional impairment (Arbabi *et al*., 2008). Of the studies, 10 investigated the treatment of TTM (Christenson *et al*., 1991; Streichenwein & Thornby, 1995; Ninan *et al*., 2000; Dougherty *et al*., 2006; Grant *et al*., 2009, 2014, 2019; Van Ameringen *et al*., 2010; Bloch *et al*., 2013; Leppink *et al*., 2017), two investigated SPD (Arbabi *et al*., 2008; Grant *et al*., 2016), two investigated both TTM and SPD (Grant *et al*., 2022, 2023b), and one investigated BDD (Phillips *et al*., 2002). No trials were identified that investigated medication for treatment of HD or other BFRBs.

The mean age of participants in the 14 trials that reported this variable was between 31.07 ± 11.51 years, including a single trial conducted in adolescents (Bloch *et al*., 2013). The median mean age of the 14 adult studies was 31.77 years, ranging from 28.1 (Grant *et al*., 2019) to 34.8 years (Grant *et al*., 2016). There was a female predominance across studies, ranging from 56% (Ninan *et al*., 2000) to 95% (Grant *et al*., 2019), with 10 trials reporting 80% or higher proportion of female participants (Christenson *et al*., 1991; Streichenwein & Thornby, 1995; Grant *et al*., 2009, 2014, 2016, 2019, 2022, 2023b; Bloch *et al*., 2013; Leppink *et al*., 2017).

With regards to psychiatric comorbidities, five trials reported comorbidity rates for the study cohort as a whole (Christenson *et al*., 1991; Grant *et al*., 2014, 2016, 2019; Leppink *et al*., 2017). For placebo groups, rates of comorbidities were reported separately in seven of the 15 trials (Streichenwein & Thornby, 1995; Phillips *et al*., 2002; Grant *et al*., 2009, 2022, 2023b; Van Ameringen *et al*., 2010; Bloch *et al*., 2013). Comorbidity rates were not reported in the remaining three trials (Ninan *et al*., 2000; Dougherty *et al*., 2006; Arbabi *et al*., 2008). In the placebo groups, rates of comorbid depression ranged from 6.25% to 66.7% in five trials (Streichenwein & Thornby, 1995; Phillips *et al*., 2002; Bloch *et al*., 2013; Grant *et al*., 2019, 2023b). Comorbid anxiety ranged from 16% to 81.25% in six trials (Streichenwein & Thornby, 1995; Van Ameringen *et al*., 2010; Bloch *et al*., 2013; Grant *et al*., 2019, 2022, 2023b). Other comorbid OCRDs ranged from 0% to 77% in three trials (Bloch *et al*., 2013; Grant *et al*., 2019, 2023b). Comorbid OCD ranged from 0% to 30.3% in five trials (Streichenwein & Thornby, 1995; Phillips *et al*., 2002; Bloch *et al*., 2013; Grant *et al*., 2016, 2023b). Comorbid ADHD ranged from 4% to 25% in three trials (Bloch *et al*., 2013; Grant *et al*., 2019, 2022). Other comorbidities were reported, but were not of interest to the outcomes of this study. Where it was only stated that there was a comorbid mood disorder, or any psychiatric comorbidity without further differentiation, these are not reported here.

#### Interventions

The 15 studies included five trials of serotonin transporter inhibitors (three fluoxetine (Christenson *et al*., 1991; Streichenwein & Thornby, 1995; Phillips *et al*., 2002), one citalopram (Arbabi *et al*., 2008), and one sertraline (Dougherty *et al*., 2006)); three trials of NAC, a glutamate modulator (Grant *et al*., 2009, 2016; Bloch *et al*., 2013); one trial of clomipramine, a mixed receptor antagonist with predominantly serotonergic activity (Ninan *et al*., 2000); one trial of memantine, an NMDA receptor antagonist (Grant *et al*., 2023b); one trial of olanzapine, a dopamine antagonist (Van Ameringen *et al*., 2010); one trial of naltrexone, an opioid antagonist (Grant *et al*., 2014); one trial of dronabinol, a cannabinoid (Grant *et al*., 2022); one trial of inositol, a carbocyclic sugar involved in cell signal transduction (Leppink *et al*., 2017); and one trial of silymarin, an antioxidant milk thistle extract (Grant *et al*., 2019). All placebo groups received a pill placebo, although it was not stated whether the placebo had any intended physiological effects (i.e., whether they were active or passive pill placebos). Two of the three crossover trials investigated fluoxetine and utilised a five-week washout period between fluoxetine and placebo (Christenson *et al*., 1991; Streichenwein & Thornby, 1995). The third crossover trial investigated silymarin and utilised a one-week washout period (Grant *et al*., 2019).

#### Outcomes

The studies measured outcomes using an average of seven (mean = 7.13 ± 1.60) scales, ranging from four (Arbabi *et al*., 2008) to 10 (Van Ameringen *et al*., 2010) scales per trial. Dichotomous measures of treatment response include the CGI-I (Grant *et al*., 2009, 2014, 2016, 2019, 2022, 2023b; Bloch *et al*., 2013; Ninan *et al*., 2000; Phillips *et al*., 2002; Van Ameringen *et al*., 2010; Leppink *et al*., 2017) and a greater than 50% reduction on the MGH-HPS (Grant *et al*., 2009). Continuous measures of symptom severity include the CGI-I (Van Ameringen *et al*., 2010) and severity (CGI-S) scales (Van Ameringen *et al*., 2010; Grant *et al*., 2014, 2016, 2019, 2009; Leppink *et al*., 2017), the YBOCS (Arbabi *et al*., 2008) and versions modified for neurotic excoriation (NE-YBOCS) (Grant *et al*., 2016), BDD (BDD-YBOCS) (Phillips *et al*., 2002) and TTM (TTM-YBOCS) (Van Ameringen *et al*., 2010), the MGH-HPS (Dougherty *et al*., 2006; Grant *et al*., 2009, 2014, 2019; Van Ameringen *et al*., 2010; Bloch *et al*., 2013; Leppink *et al*., 2017) and a modified version for skin-picking (MGH-HPS/SPD) (Grant *et al*., 2022, 2023b), the National Institute of Mental Health (NIMH) scales for BDD (BDD-NIMH) (Phillips *et al*., 2002), BFRBs (NIMH-BFRB) (Grant *et al*., 2022), TTM impairment (NIMH-TIS) (Ninan *et al*., 2000), TTM symptom severity (NIMH-TSS) (Ninan *et al*., 2000; Bloch *et al*., 2013; Grant *et al*., 2014, 2019; Leppink *et al*., 2017) and a modified version for SPD (NIMH-TSS/SPD) (Grant *et al*., 2023b), the Psychiatric Institute Trichotillomania Scale (PITS) (Dougherty *et al*., 2006; Grant *et al*., 2009), the Milwaukee Inventory for Styles of Trichotillomania – Child (MIST-C) (Bloch *et al*., 2013), the Trichotillomania Scale for Children-Child and Parent versions (TSC-C and TSC-P) (Bloch *et al*., 2013; Grant *et al*., 2019), the Trichotillomania Impact Scale (TIS) (Dougherty *et al*., 2006), the Skin-Picking Symptom Assessment Scale (SP-SAS) (Grant *et al*., 2016, 2023b), the Brief Psychiatric Rating Scale (BPRS) (Phillips *et al*., 2002), the Brown Assessment of Beliefs Scale (BABS) (Phillips *et al*., 2002) and a Visual Analogue Scale (VAS) (Arbabi *et al*., 2008). Two studies reported symptom severity as a composite of four clinical parameters related to hair-pulling, without the use of a standardised scale (Christenson *et al*., 1991; Streichenwein & Thornby, 1995).

Other measures assessed depression symptom severity, anxiety symptom severity, functional disability, and quality of life. These include the Beck Anxiety Inventory (BAI) (Dougherty *et al*., 2006), the Hamilton Rating Scale for Anxiety (HAM-A) (Grant *et al*., 2009, 2014, 2016, 2019, 2023b), the Multidimensional Anxiety Scale for Children (MASC) (Bloch *et al*., 2013), the State–Trait Anxiety Inventory (STAI) (Ninan *et al*., 2000), the Beck Depression Inventory (BDI) (Christenson *et al*., 1991; Streichenwein & Thornby, 1995; Ninan *et al*., 2000; Dougherty *et al*., 2006), the Children’s Depression Inventory (CDI) (Bloch *et al*., 2013), the Hamilton Rating Scale for Depression (HAM-D) (Christenson *et al*., 1991; Streichenwein & Thornby, 1995; Phillips *et al*., 2002; Dougherty *et al*., 2006; Grant *et al*., 2009, 2014, 2016, 2019, 2023b), the Global Assessment of Functioning (GAF) (Phillips *et al*., 2002), the Social and Occupational Functioning Scale (SOFAS) (Phillips *et al*., 2002), the Dermatology Quality of Life Index (DLQI) (Arbabi *et al*., 2008), the Quality of Life Enjoyment and Satisfaction Questionnaire (Q-LES-Q) (Dougherty *et al*., 2006; Van Ameringen *et al*., 2010), the Quality of Life Inventory (QoLI) (Grant *et al*., 2009, 2014, 2016, 2023b), the Sheehan Disability Scale (SDS) (Grant *et al*., 2009, 2014, 2016, 2019, 2022, 2023b), and the General Health Questionnaire (GHQ) (Arbabi *et al*., 2008).

### Risk of bias of included studies

**Figure 2.**
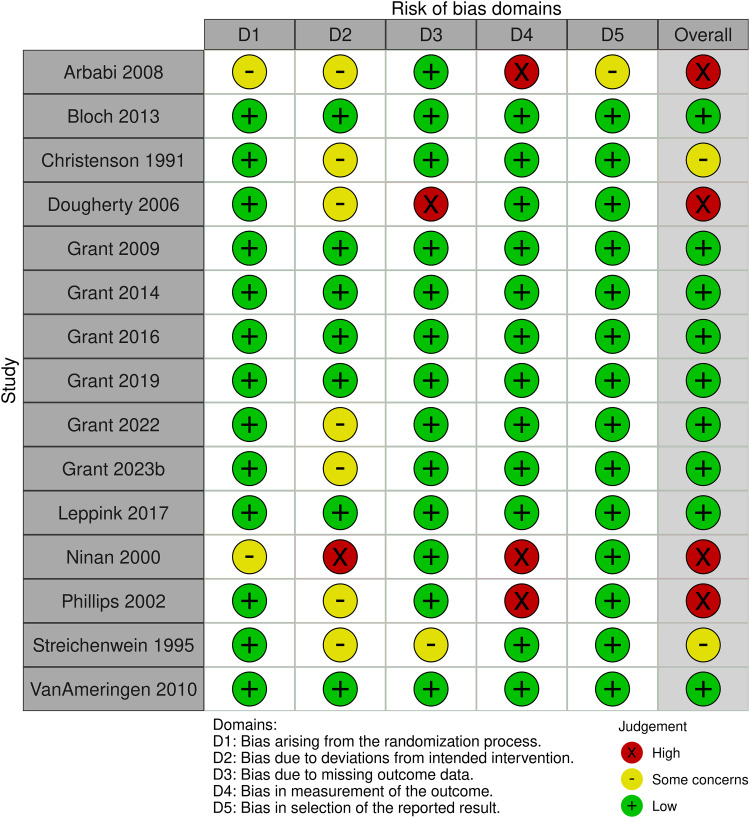
Risk of bias assessment visual summary. Domain-level and overall risk of bias assessed using the Cochrane Risk of Bias 2 tool (D1–D5). Green (+) = low risk; yellow (−) = some concerns; red (x) = high risk. Overall judgements are shown in the final column.

Risk of bias assessments are visually summarised in Figure 2. The included studies were assessed across the 5 domains below:

#### Domain 1: bias arising from the randomisation procedure

The randomisation procedure risk of bias assessment addresses concerns around allocation sequence randomisation and allocation concealment, which may lead to selection bias. This domain was rated as *low* for 13 of the 15 studies (Christenson *et al*., 1991; Streichenwein & Thornby, 1995; Phillips *et al*., 2002; Dougherty *et al*., 2006; Grant *et al*., 2009, 2014, 2016, 2019, 2022, 2023b; Van Ameringen *et al*., 2010; Bloch *et al*., 2013; Leppink *et al*., 2017). Two trials stated a randomised, double-blind study design, however further details were unavailable. These were rated as *some concerns* (Ninan *et al*., 2000; Arbabi *et al*., 2008). There were no trials at *high* risk of bias in this domain.

#### Domain 2: bias due to deviations from intended interventions

This domain relates to participant and outcome assessor blinding during the trials, and whether an appropriate analysis was conducted to estimate the effect of assignment to intervention (i.e., an ITT analysis incorporating all participants randomised). Seven of the trials were rated as *low* risk of bias (Grant *et al*., 2009, 2014, 2016, 2019; Van Ameringen *et al*., 2010; Bloch *et al*., 2013; Leppink *et al*., 2017). Seven trials were rated as having *some concerns* due to lack of utilising ITT analysis, or due to risk of possible unblinding (Christenson *et al*., 1991; Streichenwein & Thornby, 1995; Phillips *et al*., 2002; Dougherty *et al*., 2006; Arbabi *et al*., 2008; Grant *et al*., 2022, 2023b). One trial was rated as *high* risk of bias due to concerns regarding participant unblinding due to anticholinergic side effects of clomipramine (Ninan *et al*., 2000).

#### Domain 3: bias due to missing outcome data

Attrition bias arises from a large proportion of participants dropping out from a trial, when the reason for dropping out is not accounted for or when there is a significant difference in dropout rate between intervention and placebo groups. Risk of bias was *low* for 13 of the trials (Christenson *et al*., 1991; Ninan *et al*., 2000; Phillips *et al*., 2002; Arbabi *et al*., 2008; Van Ameringen *et al*., 2010; Grant *et al*., 2009, 2014, 2016, 2019, 2022, 2023b; Bloch *et al*., 2013; Leppink *et al*., 2017). One trial had *some concerns* due to attrition rate and absence of explanation for this (Streichenwein & Thornby, 1995). One trial was rated as *high* risk of bias due to high attrition rate, due to absence of explanation and differences between the intervention and control groups (Dougherty *et al*., 2006).

#### Domain 4: bias in measurement of the outcome

The outcome measurement tools used, and unblinding of outcome assessors may introduce observer or detection bias. Twelve of the trials were rated as *low* risk of bias (Christenson *et al*., 1991; Streichenwein & Thornby, 1995; Dougherty *et al*., 2006; Grant *et al*., 2009, 2014, 2016, 2019, 2022, 2023b; Van Ameringen *et al*., 2010; Bloch *et al*., 2013; Leppink *et al*., 2017). Three trials were rated as *high* risk of bias due to use of a non-standardised scale (Arbabi *et al*., 2008) or due to concerns around outcome assessor unblinding (Ninan *et al*., 2000; Phillips *et al*., 2002).

#### Domain 5: bias in selection of the reported result

Deviations from published protocols, or predetermined data analysis plans, may lead to reporting bias in trials. This domain was rated as *low* risk of bias for 14 of the trials (Christenson *et al*., 1991; Streichenwein & Thornby, 1995; Ninan *et al*., 2000; Phillips *et al*., 2002; Dougherty *et al*., 2006; Grant *et al*., 2009, 2014, 2016, 2019, 2022, 2023b; Van Ameringen *et al*., 2010; Bloch *et al*., 2013; Leppink *et al*., 2017). One trial was rated as having *some concerns* due to unavailability of published protocol and lack of detail regarding analysis plan in the published article (Arbabi *et al*., 2008).

#### Overall risk of bias

Of the 15 included trials, nine were rated as having overall *low* risk of bias (Grant *et al*., 2009, 2014, 2016, 2019, 2022, 2023b; Bloch *et al*., 2013; Van Ameringen *et al*., 2010; Leppink *et al*., 2017). Two trials were rated as having *some concerns* (Christenson *et al*., 1991; Streichenwein & Thornby, 1995). Four trials were rated as having *high* risk of bias due to the inclusion of one more domains rated as being *high* risk of bias (Ninan *et al*., 2000; Phillips *et al*., 2002; Dougherty *et al*., 2006; Arbabi *et al*., 2008).

### Placebo effect size

Meta-analysis for placebo effect size was conducted for 12 of the 15 studies for which sufficient data was available (Ninan *et al*., 2000; Phillips *et al*., 2002; Dougherty *et al*., 2006; Grant *et al*., 2009, 2014, 2016, 2019, 2022, 2023b; Van Ameringen *et al*., 2010; Bloch *et al*., 2013; Leppink *et al*., 2017). There were 35 different, eligible, outcome measurement scales for symptom severity employed (*k* = 12, mean = 2.91 ± 1.38). MGH-HPS, or the version modified for SPD, was used for nine trials (Dougherty *et al*., 2006; Grant *et al*., 2009, 2014, 2019, 2022, 2023b; Van Ameringen *et al*., 2010; Bloch *et al*., 2013; Leppink *et al*., 2017). Versions of the YBOCS were used for two trials (Phillips *et al*., 2002; Grant *et al*., 2016). The NIMH-TSS was used for the remaining trial (Ninan *et al*., 2000).

**Figure 3.**
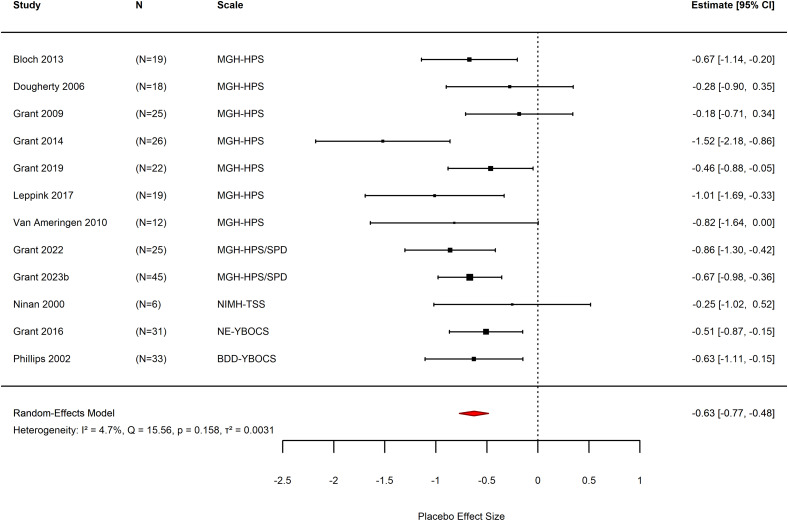
Forest plot of the effect sizes of placebo effect for the included studies. Studies are listed according to outcome scales used. A negative effect size represents reduction in symptom severity from pre- to post- measurements. BDD-YBOCS - Yale-Brown Obsessive Compulsive Scale modified for Body Dysmorphic Disorder, MGH-HPS – Massachusetts General Hospital Hair-Pulling Scale, MGH-HPS/SPD - Massachusetts General Hospital Hair-Pulling Scale and version for Skin-Picking, NE-YBOCS - Yale-Brown Obsessive Compulsive Scale modified for Neurotic Excoriation, NIMH-TSS - National Institute of Mental Health - Trichotillomania Severity Scale.

The overall pooled placebo effect size showed a moderate placebo effect (*k* = 12, SMC = −0.63, 95% CI −0.77 to −0.48, *p* < 0.0001, *N* = 281, *Z* = −8.55, Figure 3). Effect sizes in individual studies ranged from SMC = −1.52 (*N* = 26, 95% CI −2.18 to −0.86) (Grant *et al*., 2014) to SMC = −0.25 (*N* = 6, 95% CI −1.02 to 0.52) (Ninan *et al*., 2000). Heterogeneity was low, with a non-significant Chi^2^ test (*Q* = 15.56, df = 11, *p* = 0.16). Total heterogeneity was rated as *I* ^2^ = 4.73%.

The ‘leave-one-out’ sensitivity analysis demonstrates robustness of the effect size estimate, with effect sizes in the analysis ranging from SMC = −0.58 (95% CI −0.72 to −0.44) to SMC = −0.66 (95% CI 0.79 to −0.52). Removal of the study with the largest effect size resulted in very low heterogeneity (*I* ^2^ = 0%), suggesting it’s a potential outlier (Grant *et al*., 2014). Overall heterogeneity ranged from *I* ^2^ = 0% to 29%, suggesting low levels of heterogeneity.

Sensitivity analysis of the correlation coefficient (CI) in study-level SMC calculations showed a large range of effect sizes from SMC = −0.48 ± 0.17 when *r* = 0.1, up to −1.14 ± 0.49 when *r* = 0.9. This suggests a large effect of the assumed *r* value on the validity of the meta-analytic result for placebo effect size.

Subgroup analysis was conducted for methodological and clinical factors. For methodological factors, analysis of all parallel arm trials, excluding crossover trials, had a placebo effect size of SMC = −0.66 (*k* = 11, 95% CI −0.80 to −0.52, *p* < 0.001) (Ninan *et al*., 2000; Phillips *et al*., 2002; Dougherty *et al*., 2006; Van Ameringen *et al*., 2010; Bloch *et al*., 2013; Grant *et al*., 2014, 2016, 2019, 2022, 2023b; Leppink *et al*., 2017). Crossover trials could not be analysed as a separate subgroup, as only a single crossover trial had sufficient data for inclusion (Grant *et al*., 2009). Studies using a placebo run-in were not included in subgroup analysis due to insufficient number of trials (minimum of three required) (Phillips *et al*., 2002; Dougherty *et al*., 2006). Those without a placebo run-in had an effect size of SMC = −0.66 (*k* = 10, 95% CI −0.84 to −0.48, *p* < 0.001) (Ninan *et al*., 2000; Grant *et al*., 2009, 2014, 2016, 2019, 2022, 2023b; Van Ameringen *et al*., 2010; Bloch *et al*., 2013; Leppink *et al*., 2017). Studies with evidence of industry funding had an SMC = −0.38 (*k* = 3, 95% CI −0.65 to −0.11, *p* = 0.005) (Dougherty *et al*., 2006; Grant *et al*., 2009, 2016), whereas those without evidence of industry funding had an SMC = −0.72 (*k* = 9, 95% CI −0.88 to −0.55, *p* < 0.001) (Ninan *et al*., 2000; Phillips *et al*., 2002; Van Ameringen *et al*., 2010; Bloch *et al*., 2013; Grant *et al*., 2014, 2019, 2022, 2023b; Leppink *et al*., 2017). Studies at high risk of bias had showed SMC = −0.45 (*k* = 3, 95% CI −0.79 to −0.11, *p* = 0.010) (Ninan *et al*., 2000; Phillips *et al*., 2002; Dougherty *et al*., 2006), and those at low risk of bias had SMC = −0.68 (*k* = 9, 95% CI −0.87 to −0.49, *p* < 0.001) (Grant *et al*., 2009, 2014, 2016, 2019, 2022, 2023b; Van Ameringen *et al*., 2010; Bloch *et al*., 2013; Leppink *et al*., 2017).

For clinical factors, studies conducted in participants with TTM had SMC = −0.63 (k = 8, 95% CI −0.93 to −0.32, *p* < 0.001) (Ninan *et al*., 2000; Dougherty *et al*., 2006; Grant *et al*., 2009, 2014, 2019; Van Ameringen *et al*., 2010; Bloch *et al*., 2013; Leppink *et al*., 2017). Subgroup analysis was not possible for other diagnostic categories due to insufficient number of trials (required minimum of three trials per group) (Phillips *et al*., 2002; Grant *et al*., 2022, 2023b). For medication, studies investigating NAC had an SMC = −0.48 (*k* = 3, 95% CI −0.73 to −0.23) (Grant *et al*., 2009, 2016; Bloch *et al*., 2013). Studies investigating serotonin transporter inhibitors were not analysed through subgroup analysis due to insufficient data available (Phillips *et al*., 2002; Dougherty *et al*., 2006).

### Placebo response rate

**Figure 4.**
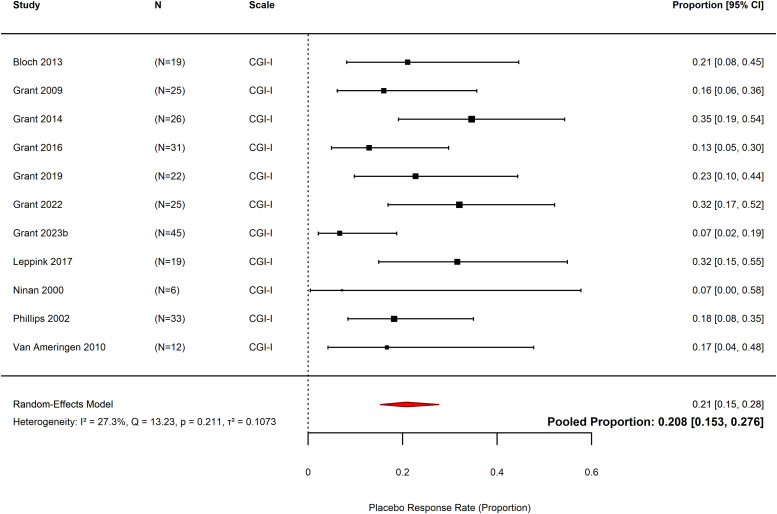
Forest plot of the placebo response rate for included studies. Placebo response is represented as the proportion of participants who were classified as treatment responders according to the outcome scale (CGI-I for all studies), where 1.00 represents 100% response rate. CGI-I – Clinical Global Impressions – Improvement Scale.

Pooled placebo response rate in 11 included studies across 212 participants was 21% (pooled proportion = 0.21, *k* = 11, *N* = 212, 95% CI 0.15 to 0.276, *p* < 0.0001, Figure 4) (Ninan *et al*., 2000; Phillips *et al*., 2002; Grant *et al*., 2009, 2014, 2016, 2019, 2022, 2023b; Van Ameringen *et al*., 2010; Bloch *et al*., 2013; Leppink *et al*., 2017). The response rate in individual studies ranged from 7% (0.07, *N* = 6, 95% CI 0.00 to 0.58) (Ninan *et al*., 2000) to 35% (pooled proportion = 0.35, *N* = 26, 95% CI 0.19 to 0.54) (Grant *et al*., 2014). All trials measured response rate using the CGI-I. Heterogeneity was low, with a non-significant Chi^2^ test (*Q* = 13.23, df = 10, *p* = 0.21). Total heterogeneity was rated as *I* ^2^ = 27.3%.

### Correlates of placebo effect size: univariate and multiple meta-regression

Univariate meta-regression was conducted to examine the effect of methodological and clinical parameters on the placebo effect size. Univariate meta-regression was conducted for primary OCRD diagnosis, year of publication, number of study sites, number of treatment arms, presence or absence of placebo run-in, flexible versus fixed dose schedule, crossover versus parallel arm study design, duration of the trial, number of outcomes measured, number of participants in placebo arm, age of participants, percentage of participants with comorbidities (depression, anxiety, other OCRD, OCD, and ADHD), medication class, baseline severity (standardised using a *Z*-score normalisation method) (Cohen *et al*., 2013), active treatment effect size (calculated as SMC), industry funding and overall risk of bias score. No significant relationships were found between any of these variables and placebo effect size (*p*-values ranging from 0.407 to 0.997). Multiple linear meta-regression was therefore not undertaken. The regression coefficients and *p*-values are shown in Table 2.

**Table 2.** Univariate regression of placebo effect size moderators

Moderator	Regression coefficient (95% CI)	*P* value
Year of study	−0.02 (−0.1 to 0.059)	0.618
No. of treatment arms	0.44 (−1.607 to 2.487)	0.674
Placebo run-in	0.069 (−1.449 to 1.587)	0.929
Flexible vs fixed	−0.263 (−1.464 to 0.937)	0.667
Crossover vs parallel	−0.224 (−2.271 to 1.823)	0.83
Duration of trial	0.047 (−0.235 to 0.329)	0.743
No. of outcomes	−0.042 (−0.451 to 0.366)	0.839
No. of participants	−0.006 (−0.063 to 0.051)	0.84
Age of participants	0 (−0.1 to 0.099)	0.997
Diagnosis of TTM	−0.455 (−1.655 to 0.745)	0.458
Diagnosis of TTM/SPD	0.203 (−1.315 to 1.721)	0.793
Diagnosis of SPD	0.44 (−1.607 to 2.487)	0.674
Diagnosis of BDD	0.515 (−1.532 to 2.562)	0.622
Comorbid depression	0 (−0.058 to 0.058)	0.991
Comorbid anxiety	0.014 (−0.122 to 0.15)	0.839
Comorbid other OCRD	0.003 (−0.029 to 0.035)	0.867
Comorbid OCD	−0.002 (−0.087 to 0.083)	0.967
Medication class (NAC)	−0.642 (−2.161 to 0.876)	0.407
Medication class (SSRI)	0.069 (−1.449 to 1.587)	0.929
Baseline severity	−0.096 (−0.891 to 0.699)	0.813
Active group effect size	−0.004 (−1.355 to 1.347)	0.996
Industry funding	−0.071 (−1.378 to 1.236)	0.915
Risk of bias	−0.219 (−1.526 to 1.087)	0.742

BDD, body dysmorphic disorder, CI, confidence interval, NAC, N-acetylcysteine, OCD, obsessive–compulsive disorder, OCRD, obsessive-compulsive related disorder, SPD, skin-picking disorder, SSRI, selective serotonin reuptake inhibitor, TTM, trichotillomania.

### Nocebo response rate

**Figure 5.**
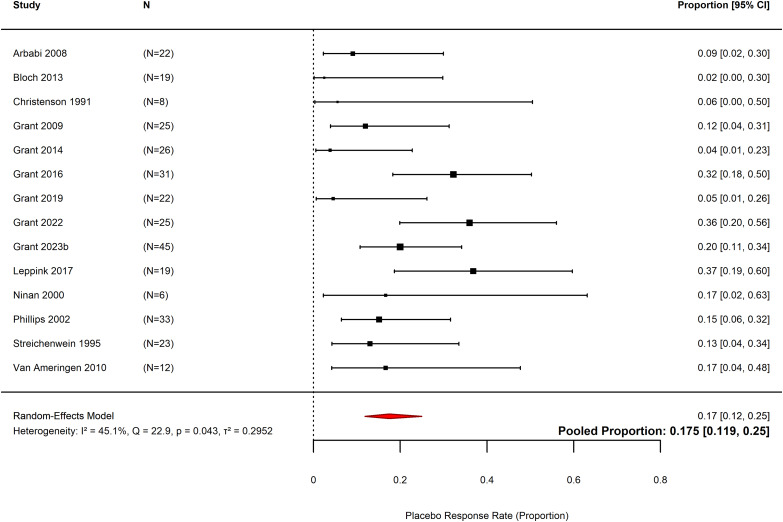
Forest plot of the nocebo response rate for included studies. Nocebo response is represented as the proportion of placebo participants who dropped out from the included studies, where 1.00 represents 100% dropout rate.

Pooled nocebo response rate in 14 included studies across 315 participants was 18% (pooled proportion = 0.17, *k* = 14, *N* = 315, 95% CI 0.12 to 0.25, *p* < 0.0001, Figure 5) (Christenson *et al*., 1991; Streichenwein & Thornby, 1995; Ninan *et al*., 2000; Phillips *et al*., 2002; Arbabi *et al*., 2008; Grant *et al*., 2009, 2014, 2016, 2019, 2022, 2023b; Van Ameringen *et al*., 2010; Bloch *et al*., 2013; Leppink *et al*., 2017). The dropout rate in individual studies ranged from 2% (0.02, *N* = 19, 95% CI 0.00 to 0.30) (Bloch *et al*., 2013) to 37% (0.37, *N* = 19, 95% CI 0.19 to 0.60) (Leppink *et al*., 2017). For all trials, all cause dropout was used to represent the number of dropouts in the placebo arms. There may be moderate heterogeneity within the sample, with a significant Chi^2^ test (*Q* = 22.9, df = 13, *p* = 0.04). Total heterogeneity was rated as *I* ^2^ = 45.1%.

### Tolerability and side effects

Two studies reported overall side effect rate, with 29% of participants experiencing side effects in these two trials (k = 2, *N* = 70) (Grant *et al*., 2022, 2023b). Overall, it was unclear if a single individual reported multiple side effects, thus side effects are reported as a proportion, reflecting how many side effects were reported per total participants. There were a total of 140 side effects reported for 315 participants in the placebo arms of 14 trials, with an average of 0.44 side effects per participant (Christenson *et al*., 1991; Streichenwein & Thornby, 1995; Ninan *et al*., 2000; Phillips *et al*., 2002; Arbabi *et al*., 2008; Grant *et al*., 2009, 2014, 2016, 2019, 2022, 2023b; Van Ameringen *et al*., 2010; Bloch *et al*., 2013; Leppink *et al*., 2017). One trial did not report specific side effects (Dougherty *et al*., 2006). A wide range of side effects were reported, affecting all major organ systems (nervous, gastrointestinal, cardiovascular, genitourinary, and respiratory systems).

Nervous system side effects included brain fog, memory impairment, insomnia, hypersomnolence, yawning, nightmares, paranoia, feelings of doom, irritability, feeling ‘high’, anxiety, depression, tremor, sweating, hot flashes, weakness, fatigue, headache, and dry mouth. Grouped together, these were reported in 75 instances in 13 trials (0.26 side effects per participant, *k* = 13, *N* = 290) (Christenson *et al*., 1991; Streichenwein & Thornby, 1995; Ninan *et al*., 2000; Phillips *et al*., 2002; Arbabi *et al*., 2008; Van Ameringen *et al*., 2010; Bloch *et al*., 2013; Grant *et al*., 2014, 2016, 2019, 2022, 2023b; Leppink *et al*., 2017).

Gastrointestinal and metabolic side effects included abdominal pain, abdominal discomfort, bloating, constipation, diarrhoea, dyspepsia, flatulence, nausea, vomiting, increased appetite, decreased appetite, and weight gain. Grouped together, these were reported in 28 instances in 13 trials (0.10 side effects per participant, *k* = 13, *N* = 290) (Christenson *et al*., 1991; Streichenwein & Thornby, 1995; Ninan *et al*., 2000; Phillips *et al*., 2002; Arbabi *et al*., 2008; Grant *et al*., 2009, 2016, 2019, 2022, 2023b; Van Ameringen *et al*., 2010; Bloch *et al*., 2013; Leppink *et al*., 2017).

Cardiovascular side effects included light-headedness, dizziness, chest pain, and rapid heartbeat. These were reported in eight instances in six trials (0.05 side effects per participant, *k* = 6, *N* = 165) (Christenson *et al*., 1991; Streichenwein & Thornby, 1995; Phillips *et al*., 2002; Grant *et al*., 2016, 2022, 2023b). Genitourinary symptoms included decreased libido and irregular menses. These were reported in four instances in two trials (0.10 side effects per participant, *k* = 2, *N* = 41) (Phillips *et al*., 2002; Bloch *et al*., 2013). Respiratory side effects consisted of a single instance of coughing in one trial (0.04 side effects per participant, *k* = 1, *N* = 25) (Grant *et al*., 2009). One instance of difficulty swallowing pills was noted in a single study (0.05 side effects per participant, *k* = 1, *N* = 19) (Bloch *et al*., 2013).

### Publication bias

**Figure 6.**
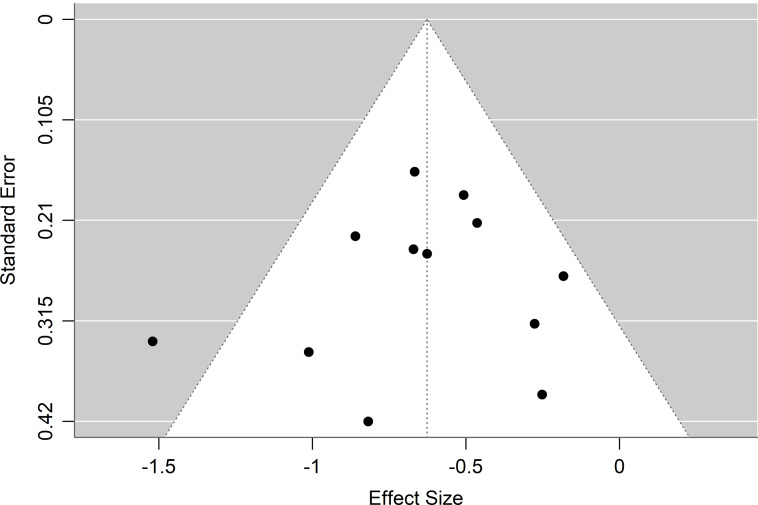
Funnel plot of the placebo effect sizes. Negative effect sizes represent improvement in symptom severity from pre- to post- measurements in the placebo groups of included studies.

Publication bias was examined through Egger’s test and visual inspection of a funnel plot for placebo effect size (Figure 6). Egger’s test showed no evidence of publication bias (*k* = 12, *p* = 0.633). Visual inspection of the funnel plot shows a relatively symmetrical distribution, with some asymmetry favouring the left side of the plot, representing larger effect sizes for pre- to post-placebo SMC. The studies with higher precision are clustered near the pooled effect size, which is representative of an unbiased dataset. Whilst there is some asymmetry, it is slight, with six studies on the left, five studies on the right, and one study in the centre of the plot. This suggests a slight overrepresentation of studies with large effect size on the left. Additionally, the slight left-skew may be indicative of a small-study effect, wherein smaller studies with smaller effect sizes may be missing. A relative paucity of studies in the lower-right region suggests a lack of smaller studies with small effect sizes, which may have not been published. The overall observed variability, however, is within the expected range.

## Discussion

### Placebo effect and its moderators

A small body of evidence is available for investigating the placebo and nocebo effects in pharmacotherapy trials for OCRDs. Fifteen RCTs in TTM, SPD, and BDD are included in this review, published between 1991 and 2023, with 640 total participants and 341 participants in the placebo arm of the trials. The overall placebo effect size for the 12 studies included in this analysis was moderate (*k* = 12, SMC = −0.63, 95% CI −0.77 to −0.48, *p* < 0.0001, *N* = 281, *Z* = −8.55, Figure 3) with low heterogeneity (*I* ^2^ = 4.73%).

This is higher than the effect size of reported in prior studies in OCD (effect size = 0.32) (Kotzalidis *et al*., 2019), but lower than reported for anxiety disorders (1.85) (Bandelow *et al*., 2015) and major depression (effect size = 1.10) (Scott *et al*., 2022). An effect size of 0.63, as found in this study, was similar to the median effect size across all psychiatric conditions reported in a recent umbrella review (median effect size = 0.64) (Huneke *et al*., 2024), and closest to that reported in schizophrenia (effect size = 0.64) (Czobor *et al*., 2022) and panic disorder (0.57) (Ahmadzad-Asl *et al*., 2022). The placebo response rate of 21% (pooled proportion = 0.21, *k* = 11, *N* = 212, 95% CI 0.15 to 0.276, *p* < 0.0001, Figure 4) is consistent with this, showing similarity to that reported in TTM (31%) and OCD (31%), and lower than reported for major depression (49.6%) and anxiety disorders (39.6%) (Cohen *et al*., 2013; Grant *et al*., 2017).

Differences in neurobiology and psychopathology between OCD, OCRDs, depression, and anxiety may partially explain the varied placebo effect size and response rate across conditions. The neurobiological mechanisms underlying the placebo response involve dopaminergic and opioid pathways, including increased striatal dopamine release and endogenous opioid release, with altered activity also noted in the orbitofrontal cortex, dorsolateral prefrontal cortex, cingulate cortex, ventral striatum, nucleus accumbens, insula, amygdala, thalamus, hypothalamus, periaqueductal grey and default mode network (Amanzio & Benedetti, 1999; Amanzio *et al*., 2001; de la Fuente-Fernández *et al*., 2001; Zubieta & Stohler, 2009; Lidstone *et al*., 2010; Burke *et al*., 2022; Huneke *et al*., 2022). OCD tends to have fewer overlaps with these regions, compared to depression and anxiety disorder, which show notable changes within the implicated limbic system regions and default mode network (Goodman *et al*., 2021; Runia *et al*., 2022).

Further, neuroimaging studies in OCRDs, including TTM, SPD, and BDD, show changes across a wide range of brain regions, notably within cortico-striatal-thalamo-cortical loops, frontal, limbic, and occipital pathways (Grace *et al*., 2019; Grant *et al*., 2023a). This demonstrates overlap between OCD, OCRDs, anxiety, and depression, potentially with greater overlap between OCRDs and other conditions compared to OCD and other conditions. These differences between neurobiological mechanisms underlying the psychopathology and overlap with regions implicated in the placebo response may account for some of the variability seen in the placebo effect size and response rate.

Methodological factors related to study design may also play a role in the observed placebo effect. Our analysis failed to demonstrate a significant relationship between any of the potential methodological moderators and the placebo effect size.

We found no correlation between placebo effect size and year of publication (1991–2023), which supports Furukawa et al.’s finding of no correlation after 1991 (Furukawa *et al*., 2018). Mohamadi et al., however, demonstrate a slight, significant increase in placebo effect size across OCD trials in the last 30 years, whilst Kotzalidis et al., demonstrated a significant net increase in placebo effect size in OCD trials from 1991 to 2017 (Kotzalidis *et al*., 2019; Mohamadi *et al*., 2023).

The medication class investigated is a potential moderator of the placebo response, as side effects may lead to unmasking of outcome assessors (Holper & Hengartner, 2020). We found no correlation between medication class and placebo response. The analysis was limited by the small number of trials for any particular medication group, investigating 11 different medications across nine medication classes, limiting the analysis of correlations due to insufficient number of studies for each subgroup. In future studies, grouping medications by their neurobiological or physiological effects may allow for further investigation of this aspect. The utilisation of an ‘active’ placebo, with physiological receptor-binding profiles designed to mimic the side effect profile of the active agent may also aid in evaluating this aspect of the placebo effect (Moncrieff *et al*., 2004). However, multiple side effects were reported in the placebo groups of the included studies, across multiple organ systems, indicating that even inert pill placebos result in adverse effects. This warrants further investigation through researching the nocebo effect.

Earlier trials in OCD investigated the efficacy of clomipramine, a TCA with anticholinergic side effects, and were predominantly conducted in treatment-naïve individuals. This indicates a potential confounder in the observed increase placebo response over time. However, this also highlights another potential moderator of the placebo response. As this was the first available pharmacological agent investigated in OCD, the treatment-naïve individuals likely had reduced expectation of response, compared to participants in more recent trials who may enter the trial with preconceived notions around treatment efficacy. Of note, multiple trials included participants who were either on other psychopharmacological treatment at the time, or who had been on treatment previously. This has an impact on the expectation of participants when entering the trial framework (Leuchter *et al*., 2014). As no studies used a measure of expectation at outset, this precluded investigation of this moderator of the placebo effect, but could be investigated in the future should trials employ a measure of expectation at enrolment.

We found no correlation between any of the methodological factors and placebo effect size. Methodological aspects such as number of study sites, number of study arms, crossover versus parallel group study design, presence or absence of placebo run-in, fixed or flexible dosing regimen, duration of the study, baseline severity, and number of outcomes measured may, however, account for non-biological sources of variability in the observed placebo effect (Huneke *et al*., 2024). The finding of no correlation may be due to the small number of included studies, missing data in some of the trials, and lack of variation in these variables across the included trials. This could be investigated further should more studies become available for analysis.

Similar to the finding of no correlation between placebo effect and methodological factors, no correlation was found between placebo effect size and clinical variables. In addition, no clinical or demographic characteristics were correlated with placebo response in a previous analysis of placebo response in TTM (Grant *et al*., 2017). Clinical factors such as specific OCRD diagnosis, baseline severity, age, gender, and psychiatric comorbidities may, however, play a role in moderating the placebo response (Huneke *et al*., 2024). The finding of no correlation in this study may be due to insufficient number of included trials to run a robust meta-regression analysis.

### Nocebo effects and side effects

Similar to the relationship between positive expectations and placebo response, negative expectations may lead to a nocebo response in clinical trials (Blasini *et al*., 2018). In our study, the pooled dropout rate was 18%, however, it was unclear how many participants dropped out due to adverse events. This is a higher rate than that reported in depression. In a meta-analysis of nocebo effects in clinical trials for depression, 4.5% of the participants discontinued placebo treatment due to intolerance (Mitsikostas *et al*., 2014).

On average, 0.44 side effects were experienced per placebo participant across 14 trials. In two trials, 29% of participants experienced at least one side effect. In a meta-analysis of nocebo effects in depression trials, 44.7% of participants were reported as experiencing at least one side effect (Mitsikostas *et al*., 2014). This indicates the presence of side effects to inert pill placebo, indicating the presence of a degree of negative expectations within the sample cohort, and warrants further investigation.

### Quality of the evidence and risk of bias

Risk of bias in the study sample was generally low, with some concerns raised for not employing an ITT analysis, lack of information around reasons for attrition, and possible unblinding in trials of fluoxetine and clomipramine (Ninan *et al*., 2000; Phillips *et al*., 2002). Levels of blinding and unblinding may be relevant to the expectation response, and could potentially impact observed placebo effect size (Huneke *et al*., 2024). In addition, lack of comparison with published protocols may have led to further bias within the included trials, although most stated analysis plans within the retrieved publications. Additionally, there was little evidence to support the presence of publication bias.

When assessing the impact of industry funding and risk of bias on placebo effect size, no correlation was found on meta-regression between the overall risk of bias score or industry funding on placebo effect size. However, subgroup analysis revealed a lower placebo effect size for trials with evidence of industry funding (SMC = −0.38, *k* = 3, 95% CI −0.65 to −0.11, *p* = 0.005) compared to those without (SMC = −0.72, *k* = 9, 95% CI −0.88 to −0.55, *p* < 0.001) and for those at high risk of bias (SMC = −0.45, *k* = 3, 95% CI −0.79 to −0.11, *p* = 0.010) compared to those at low risk of bias (SMC = −0.68, *k* = 9, 95% CI −0.87 to −0.49, *p* < 0.001). This highlights the potential for methodological factors impacting the observed placebo effect size, and impacting the reported efficacy of medication from RCTs.

## Conclusion

The findings of this systematic review and meta-analysis underscores the importance of recognising the placebo and nocebo effects when conducting and interpreting pharmacological trials of OCRDs. The moderate magnitude of effect size emphasises that clinicians and researchers should be mindful of the potential contribution of expectancy effects to treatment response and resultant efficacy results. A deeper understanding of the neurobiology of OCRDs may shed light on the interplay between psychopathology and expectancy effects.

None of the potential clinical or methodological moderators demonstrated significant effects on the observed placebo response rate or effect size. However, these require further investigation with larger samples to produce robust findings. Analysis using individual participant data may further enhance the potential for statistical enquiry. Additionally, we did not investigate the influence of clinician- versus participant-rated scales on the outcome of the analysis, nor did we investigate the influence of ITT versus completer analysis on the placebo effect or response rates. These could be investigated in future studies.

Risk of bias assessment remains paramount to the valid interpretation of trials and reporting of meta-analytic findings. Key aspects such as blinding, selective reporting, and publication bias may play an important role in understanding the impact of the placebo and nocebo effects on reported efficacy of pharmacological treatments for OCRDs.

This is the first study to systematically investigate placebo effect sizes and response rates across all pharmacology RCTs for OCRDs, and to investigate potential clinical and methodological moderators of the placebo effect in OCRDs. Further research may investigate gaps identified within this review, including the role of expectation at study onset, the role of unblinding, the specific neurobiological factors in OCRD that drive placebo and nocebo effects, investigation of ITT versus completer analyses and analysis of clinician-rated versus self-report scales.

Ultimately, insights gained from investigating expectation in clinical practice may enhance patient outcomes, reducing symptom severity through harnessing factors that promote placebo response, and minimising the risk of adverse effects to medication by reducing nocebo effects.
